# Successful treatment of long-COVID postural tachycardia syndrome with epipharyngeal abrasive therapy in an adolescent patient: A case report

**DOI:** 10.1097/MD.0000000000043333

**Published:** 2025-07-11

**Authors:** Hiroyuki Takezawa

**Affiliations:** a Takezawa ENT Clinic, Obihiro, Hokkaido, Japan.

**Keywords:** epipharyngeal abrasive therapy (EAT), long-COVID, postural tachycardia syndrome (POTS)

## Abstract

**Rationale:**

Postural orthostatic tachycardia syndrome (POTS) is a type of autonomic dysfunction that can occur following coronavirus disease (COVID-19) infection, particularly in adolescents. Treatment options are limited and often ineffective. We report a case of long COVID-associated POTS that responded favorably to epipharyngeal abrasive therapy (EAT), a treatment targeting chronic inflammation in the epipharynx.

**Patient concerns:**

A previously healthy adolescent developed persistent fatigue, headache, dizziness, insomnia, and orthostatic intolerance lasting over 3 months after a COVID-19 infection. The symptoms severely impaired daily functioning, rendering the patient bedridden.

**Diagnoses:**

Based on clinical symptoms and a positive orthostatic test showing excessive postural tachycardia without hypotension, the patient was diagnosed with POTS associated with long COVID. Chronic epipharyngitis was also noted upon endoscopic examination.

**Interventions:**

The patient underwent weekly sessions of EAT for a total of 120 days. No additional pharmacologic treatment was provided during this period.

**Outcomes:**

The patient showed marked improvement in symptoms, including increased tolerance to standing, reduced fatigue, and improved sleep quality. Objective improvement was confirmed through repeat orthostatic testing. The patient resumed school attendance and daily activities without limitation.

**Lessons:**

This case highlights the potential effectiveness of EAT in treating long COVID-related POTS in adolescents. EAT may offer a nonpharmacological treatment option by addressing underlying chronic epipharyngeal inflammation, a possible contributor to autonomic dysfunction.

## 1. Introduction

Postural tachycardia syndrome (POTS) has various underlying causes, and its treatment has not yet been established. Recently, long-COVID has garnered significant attention, and it is known that POTS can develop following coronavirus disease (COVID-19). Since the emergence of COVID-19, a growing number of patients have reported persistent symptoms that meet the diagnostic criteria for POTS, even after recovery from the acute phase of infection. Recent studies suggest that POTS may develop in approximately 10% to 30% of individuals with long COVID, particularly among young women.^[1–3]^ However, no standardized treatment has been reported to date. We report a case of post-COVID POTS in which we implemented epipharyngeal abrasive therapy (EAT) to improve autonomic dysfunction and observed a remarkable improvement. EAT is a procedure in which the nasopharynx is visualized using an endoscope, and a cotton swab soaked in 1% zinc chloride solution is inserted transnasally from the contralateral side to abrade the nasopharyngeal mucosa. This therapy aims to reduce nasopharyngeal inflammation and improve autonomic dysfunction through vagus nerve stimulation. EAT has been utilized in Japan for more than 50 years.

## 2. Case presentation

A 16-year-old boy had been functioning well in daily life, attending school, and engaging in extracurricular activities without any impairment. However, after contracting COVID-19 in May 2022, he developed persistent fatigue, memory impairment, dizziness, headaches, and insomnia, which prevented him from performing these activities. Attempts to attend school exacerbated his symptoms and eventually confined him to bed. Four months postinfection, the patient became nearly completely bedridden and was unable to attend school. Despite a comprehensive evaluation at a local general hospital, brain MRI, neurological examinations, and blood tests relevant to the differential diagnosis of ME/CFS revealed no abnormalities. He was prescribed medications such as midodrine (4 mg/day) from November 2022 to May 2023 for orthostatic dysregulation.

When symptoms persisted until May 2023, he sought treatment at our clinic specializing in long-COVID. The possibility of myalgic encephalomyelitis/chronic fatigue (ME/CFS) and orthostatic hypotension was considered, and an orthostatic test was performed. Additional tests, including thyroid function, cortisol levels, and brain magnetic resonance imaging, were conducted by a previous physician, and no abnormalities were detected. Cerebrospinal fluid (CSF) examination was not performed because no neurological abnormalities were observed. Additionally, 24-hour ambulatory blood pressure and heart rate monitoring were not conducted. The patient had no notable medical history prior to COVID-19. Fatigue and headache caused by long-COVID, as well as ME/CFS, were considered based on the symptoms. However, the orthostatic test revealed an increase in pulse rate, meeting the diagnostic criteria for POTS. In addition, the absence of hypotension further supported the diagnosis. His condition was diagnosed as long-COVID-associated POTS, and EAT was initiated. EAT is expected to ameliorate chronic epipharyngeal inflammation and potentially improve autonomic function through stimulation of the vagus nerve. At the initiation of EAT, transnasal endoscopic examination revealed inflammation of the nasopharynx. EAT was performed once a week for 15 sessions. At the start of the treatment, the patient was unable to attend school or perform daily activities because of fatigue and headaches.

### 2.1. Standing test results

The orthostatic test was conducted by measuring blood pressure and heart rate after 10 minutes in the supine position, immediately after assuming a sitting position, immediately after standing, and after 10 minutes in the standing position. The evaluation was based on the differences relative to the measurements taken after 10 minutes in the supine position.

Initial visit: Upon standing, the patient demonstrated significant hemodynamic changes: blood pressure increased from 124/61 to 147/80 mm Hg, pulse pressure narrowed from 63 to 25 mm Hg, and heart rate rose markedly from 78 to 139 beats/min (Δ61 beats/min). While hypotension was absent, the pronounced heart rate increase coupled with symptom persistence for 1 year confirmed the POTS diagnosis.

After 90 days of EAT: Hemodynamic parameters showed improvement with standing: blood pressure changed from 105/51 to 108/59 mm Hg, pulse pressure decreased from 54 to 40 mm Hg, and heart rate increased from 60 to 78 beats/min (Δ18 beats/min). The patient resumed school attendance despite experiencing post-school fatigue.

After 120 days of EAT (15 sessions): Further improvements in orthostatic response were observed: blood pressure changed from 116/60 to 109/74 mm Hg, pulse pressure decreased from 56 to 40 mm Hg, and heart rate increased from 69 to 89 beats/min (Δ20 beats/min) (Table 1). With stabilized heart rate response and improved concentration, the patient fully resumed school and daily activities, leading to treatment discontinuation in December 2023.

**Table 1:** Changes in vital signs during treatment.

Parameter	Initial visit	After 90 d EAT	After 120 d EAT
Blood pressure (mm Hg)			
Supine	124/61	105/51	116/60
Standing	147/80	108/59	109/74
Pulse pressure (mm Hg)			
Change upon standing	63 → 25	54 → 40	56 → 40
Heart rate (beats/min)			
Supine	78	60	69
Standing	139	78	89
Change (Δ)	+61	+18	+20

EAT = epipharyngeal abrasive therapy.

## 3. Discussion

This case report describes a patient who had been functioning well in daily life, attending school, and engaging in extracurricular activities without impairment before contracting COVID-19. The patient did not receive any additional therapy during the EAT. After the infection, the patient developed long-COVID POTS, with symptoms persisting for approximately 1 year. The patient was bedridden during the presentation at our clinic. Following a 120-day course of EAT administered once weekly, the patient demonstrated significant improvement in symptoms and orthostatic test results.

POTS, a chronic multisystem disorder involving the autonomic nervous system, is characterized by excessive sinus tachycardia upon standing without accompanying hypotension.^[4]^ It frequently manifests during adolescence and can be triggered by immune stressors, such as viral infections, including COVID-19.^[3,5,6]^ The patient also developed fatigue, headache, insomnia, and orthostatic intolerance after COVID-19, which persisted for more than 3 months. Nasopharyngitis was also observed.

The current treatment landscape for POTS lacks FDA-approved medications, and available therapies often lack robust evidence.^[4]^ In this case, EAT, a treatment unique to Japan with a history of more than 50 years, was used. Its mechanisms of action are diverse and include alleviation of local inflammation and regulation of autonomic dysfunction.^[7]^

EAT may stimulate the vagus nerve located in the nasopharynx, reducing inflammatory cytokines such as IL-6 and TNF-α, which are implicated in long-COVID.^[7,8]^ Furthermore, EAT may directly influence autonomic balance and enhance cerebrospinal fluid drainage through the nasopharyngeal lymphatic plexus,^[8–10]^ improving symptoms of fatigue, headache, and concentration deficits associated with long-COVID.^[11]^ There have been no reports of EAT being applied specifically to treat long-COVID POTS. However, EAT has been reported to be effective in the treatment of long-COVID. Considering that our patient also presented with nasopharyngitis and autonomic dysfunction, an EAT was implemented. The present case represents postinfectious POTS that developed following COVID-19, with presumed underlying mechanisms involving autonomic dysfunction and immune-mediated cytokine activity triggered by viral infection. Recent reports have demonstrated the persistence of SARS-CoV-2 RNA and inflammatory cytokines in epipharyngeal tissue. EAT has been shown to improve the symptoms of long COVID, potentially by facilitating the removal of residual viral RNA and reducing local inflammatory cytokine expression in the epipharynx.^[12]^

Ito investigated the effects of EAT on autonomic function in patients with chronic epipharyngitis. Using the tilt table test and heart rate variability analysis, they evaluated autonomic activity before and after EAT.^[13]^ The results demonstrated a tendency toward suppression of sympathetic activity and improvement in parasympathetic function. Additionally, EAT appeared to enhance baroreceptor reflex function during tilt testing. These findings suggest that EAT may help modulate autonomic balance and serve as a potential therapeutic option for patients with autonomic dysfunction. This report provides important preliminary evidence supporting the autonomic regulatory effects of EAT.^[10]^

This case report describes a single patient without a control group, limiting generalizability and making the causal relationship between EAT and improvement uncertain. Spontaneous remission cannot be excluded. The POTS diagnosis was clinical, without comprehensive autonomic testing, which reduces the diagnostic precision. Furthermore, the mechanism of action of EAT remains unclear, and its long-term efficacy is unknown.

This case demonstrated that EAT successfully improved the symptoms and quality of life of a patient with long-COVID POTS without the need for pharmacological intervention. Our findings suggest that EAT is a potential therapeutic option for managing long-COVID POTS.

## Author contributions

**Conceptualization:** Hiroyuki Takezawa.

**Data curation:** Hiroyuki Takezawa.

**Formal analysis:** Hiroyuki Takezawa.

**Investigation:** Hiroyuki Takezawa.

**Methodology:** Hiroyuki Takezawa.

**Supervision:** Hiroyuki Takezawa.

**Validation:** Hiroyuki Takezawa.

**Writing – original draft:** Hiroyuki Takezawa.

**Writing – review & editing:** Hiroyuki Takezawa.

## References

[1] Blitshteyn S Whitelaw S. Postural orthostatic tachycardia syndrome (POTS) and other autonomic disorders after COVID-19 infection: a case series of 20 patients [published correction appears in Immunol Res. 2021;69(2):212. doi: 10.1007/s12026-021-09191-7.]. Immunol Res. 2021;69:205–11. 33786700 10.1007/s12026-021-09185-5 PMC8009458

[2] Johansson M Ståhlberg M Runold M. Long-haul post-COVID-19 symptoms presenting as a variant of postural orthostatic tachycardia syndrome: the Swedish experience. JACC Case Rep. 2021;3:573–80. 33723532 10.1016/j.jaccas.2021.01.009 PMC7946344

[3] Raj SR Arnold AC Barboi A; American Autonomic Society. Long-COVID postural tachycardia syndrome: an American autonomic society statement. Clin Auton Res. 2021;31:365–8. 33740207 10.1007/s10286-021-00798-2 PMC7976723

[4] Vernino S Bourne KM Stiles LE. Postural orthostatic tachycardia syndrome (POTS): State of the science and clinical care from a 2019 National Institutes of Health Expert Consensus Meeting – part 1. Auton Neurosci. 2021;235:102828. 34144933 10.1016/j.autneu.2021.102828 PMC8455420

[5] Davis HE McCorkell L Vogel JM Topol EJ. Long COVID: major findings, mechanisms and recommendations. Nat Rev Microbiol. 2023;21:133–46. Erratum in: Nat Rev Microbiol. 2023;21(6):408. 36639608 10.1038/s41579-022-00846-2 PMC9839201

[6] Kavi L. Postural tachycardia syndrome and long COVID: an update. Br J Gen Pract. 2021;72:8–9. 34972793 10.3399/bjgp22X718037 PMC8714530

[7] Hotta O Inoue CN Tanaka A Ieiri N. Possible mechanisms underlying epipharyngeal abrasive therapy (EAT) with ZnCl2 solution for the treatment of autoimmune diseases and functional somatic syndrome. J Antivir Antiretrovir. 2017;9:81–6.

[8] Nishi K Yoshimoto S Nishi S. Epipharyngeal abrasive therapy (EAT) reduces the mRNA expression of major proinflammatory cytokine IL-6 in chronic epipharyngitis. Int J Mol Sci. 2022;23:9205. 36012469 10.3390/ijms23169205 PMC9409341

[9] Yoon JH Jin H Kim HJ. Nasopharyngeal lymphatic plexus is a hub for cerebrospinal fluid drainage. Nature. 2024;625:768–77. 38200313 10.1038/s41586-023-06899-4 PMC10808075

[10] Ito H. Autonomic nervous system regulation effects of epipharyngeal abrasive therapy for myalgic encephalomyelitis/chronic fatigue syndrome associated with chronic epipharyngitis. Cureus. 2023;15:e33777. 36655156 10.7759/cureus.33777 PMC9840732

[11] Imai K Yamano T Nishi S. Epipharyngeal abrasive therapy (EAT) has potential as a novel method for long COVID treatment. Viruses. 2022;14:907. 35632649 10.3390/v14050907 PMC9147901

[12] Nishi K Yoshimoto S Tanaka T. Spatial transcriptomics of the epipharynx in long COVID identifies SARS-CoV-2 signalling pathways and the therapeutic potential of epipharyngeal abrasive therapy. Sci Rep. 2025;15:8618. 40074801 10.1038/s41598-025-92908-7 PMC11903674

[13] Hirobumi I. The effect of epipharyngeal abrasive therapy (EAT) on the baroreceptor reflex (BR). Cureus. 2023;15:e45080. 37705568 10.7759/cureus.45080 PMC10496426

